# A FDG-PET Study of Metabolic Networks in Apolipoprotein E ε4 Allele Carriers

**DOI:** 10.1371/journal.pone.0132300

**Published:** 2015-07-10

**Authors:** Zhijun Yao, Bin Hu, Jiaxiang Zheng, Weihao Zheng, Xuejiao Chen, Xiang Gao, Yuanwei Xie, Lei Fang, Alzheimer’s Disease Neuroimaging Initiative

**Affiliations:** 1 School of Information Science and Engineering, Lanzhou University, Lanzhou, China; 2 PET/CT Center, Affiliated Lanzhou General Hospital of Lanzhou Military Area Command, 333 South Binhe Road, Lanzhou, China; Beijing Normal University,Beijing, CHINA

## Abstract

Recently, some studies have applied the graph theory in brain network analysis in Alzheimer's disease (AD) and Mild Cognitive Impairment (MCI). However, relatively little research has specifically explored the properties of the metabolic network in apolipoprotein E (APOE) *ε*4 allele carriers. In our study, all the subjects, including ADs, MCIs and NCs (normal controls) were divided into 165 APOE *ε*4 carriers and 165 APOE *ε*4 noncarriers. To establish the metabolic network for all brain regions except the cerebellum, cerebral glucose metabolism data obtained from FDG-PET (^18^F-fluorodeoxyglu-cose positron emission tomography) were segmented into 90 areas with automated anatomical labeling (AAL) template. Then, the properties of the networks were computed to explore the between-group differences. Our results suggested that both APOE *ε*4 carriers and noncarriers showed the small-world properties. Besides, compared with APOE *ε*4 noncarriers, the carriers showed a lower clustering coefficient. In addition, significant changes in 6 hub brain regions were found in between-group nodal centrality. Namely, compared with APOE *ε*4 noncarriers, significant decreases of the nodal centrality were found in left insula, right insula, right anterior cingulate, right paracingulate gyri, left cuneus, as well as significant increases in left paracentral lobule and left heschl gyrus in APOE *ε*4 carriers. Increased local short distance interregional correlations and disrupted long distance interregional correlations were found, which may support the point that the APOE *ε*4 carriers were more similar with AD or MCI in FDG uptake. In summary, the organization of metabolic network in APOE *ε*4 carriers indicated a less optimal pattern and APOE *ε*4 might be a risk factor for AD.

## Introduction

Alzheimer’s disease (AD) is considered with progressive memory loss and cognitive decline. There are some established genetic risk factors and non-genetic environmental factors about the AD. The *ε*4 allele of apolipoprotein E (APOE) might be the strongest genetic risk factor [1, 2]. For instance, the APOE4-induced BBB (blood-brain barrier) anomalies was found in a study on the relationship between cerebrovascular defects and AD, which may be associated with the development in AD [3]. The frequency of the *ε*4 allele of apolipoprotein E (APOE) was suggested in a high level in northern regions of Europe and *ε*4 allele existed in roughly 20–25% of North Americans and Europeans[4, 5]. This result provided the support to study the individuals with apolipoprotein E (APOE) in a large sample size[5]. A univariate logistic regression analysis found that APOE *ε*4 was significantly related to AD predictability [6].

As an imaging biomarker, PET with ^18^F-fluorodeoxyglu-cose (FDG) could predict future Alzheimer’s disease (AD) or mild cognitive impairment (MCI) [7, 8]. A recent study showed that the distribution of amyloid plaques and glucose metabolism were affected differentially by the APOE genotype [9]. Besides, in FDG-PET and magnetic resonance imaging (MRI) measurements, APOE *ε*4 gene dose was significantly associated with regional glucose metabolism and regional gray matter [10]. The APOE *ε*4 homozygote group also showed greater whole brain atrophy rate than noncarriers [11].

Recently, the graph theoretical analysis has been applied to study the topological organization of complex brain network and the metabolic brain network analysis was used to study patients with Parkinson’s disease [12–14]. However, less research existed on the metabolic brain network of the *ε*4 allele of apolipoprotein E (APOE) carriers [15]. In this paper, in order to explore the relationship between APOE *ε*4 and the risk of AD progression, we established the PET metabolic brain network to explore the differences of brain network properties between APOE *ε*4 carriers and noncarriers. We hypothesized that APOE *ε*4 allele would be correlated to the risk of AD progression.

## Methods and Subjects

### Ethics Statement

We used Alzheimer’s Disease Neuroimaging Initiative (ADNI) subject data gathered over 50 sites from the United States and Canada. The Principle Investigator (lead author) of this initiative is Michael W. Weiner, MD, UC San Francisco (Email ADNI: adni@loni.usc.edu). All subjects in ADNI had signed written informed consent for the enrollment of imaging and genetic data collection and completed questionnaires. The data collections were approved by every participating site’s Institutional Review Board (IRB). The complete list of ADNI sites’ IRBs can be found in the supporting information file (S1 File). All authors confirm that the named institutional review board or ethics committee specifically approved the ADNI study and the data was only analyzed.

### Subjects

All of the FDG-PET data were downloaded from the Alzheimer’s Disease Neuroimaging Initiative (ADNI) database (http://www.loni.ucla.edu/ADNI/). The major goal of ADNI was to lead a uniform standard in multi-site MRI and PET data with AD and MCI. We pooled all subjects including MCI, AD and normal controls and divided into 165 APOE *ε*4 carriers and 165 noncarriers. The 165 APOE *ε*4 carriers (20ADs, 75MCIs, 70NCs) with age ranging from 62 to 91 (M = 74.18; SD = 6.695) (female/male, 81:84) and 165 noncarriers (32 ADs, 52 MCIs, 81 NCs) with age from 57 to 95 (M = 75.58; SD = 7.011) (female/male, 84:81). There is no significant between-group difference in the age (p = 0.0635 >0.05) and education (p = 0.1989 >0.05) by 2-sample t test.

According to the previous studies by *Morra et al*. and *Jie Shi et al*., the heterozygous APOE ε4 carriers and homozygous APOE ε4 carriers (ε2/ε4 N = 8, ε3/ε4 N = 124, ε4/ε4 N = 33) were chosen to form the APOE ε4 carriers group[5, 16–18]. The APOE ε4 noncarriers group included heterozygous noncarriers (ε2/ε3 N = 37, ε2/ε2 N = 1) and homozygous noncarriers (ε3/ε3 N = 137).

Criteria for ADNI eligibility and diagnostic classifications are instructed at http://www.adni-info.org/Scientists/ADNIGrant/ProtocolSummary.aspx.

### FDG-PET Data Acquisition and Processing

All FDG-PET images were co-registered, averaged, normalized (standardized image and voxel size) and smoothed to a uniform resolution (8 mm full-width at half-maximum). The PET scans needed dynamic 30-min, six frame (5min each) acquisitions starting 30 min post ^18^F-labelled fluoro-deoxy-glucose (^18^F-FDG) injection. We normalized all images spatially to the PET Montreal Neurological Institute brain space template, scaled, and averaged using SPM8 (Statistical Parametric Mapping 8) running under Matlab 7.11 on the CentOS 6.5 and these images were acquired using Siemens, GE and Philips PET scanners in resting state. The spatial normalization included a 12-parameter affine transformation and this process was followed by nonlinear iterative spatial transformation through SPM8.

### Establishment of the metabolic networks

In this study, the functional connections in metabolic networks were considered as statistical correlations between pairs of average metabolic values in corresponding AAL areas. We defined an existence of functional connection in pair of brain areas as the correlation coefficient between the pair of brain areas was statistically significant [19]. According to the previous analysis, a linear regression was introduced to remove the effects of age, gender and whole brain metabolic level on all subjects’ measurements in every AAL brain region[20]. Then, an interregional correlation matrix (90×90) was calculated by computing the partial correlation coefficients across subjects between the average metabolic values in each pair of AAL brain areas. At last, the entire number of the whole interregional correlations in AAL brain areas was 90×89 / 2 = 4005 [19].

### Graph theoretical methods

A uniform correlation threshold was used to find the abnormal changes of the networks between APOE *ε*4 carriers and noncarriers, with construction of the binarized matrices Pij (M nodes and L edges) [19, 21]. Entire nodes and edges had their corresponding AAL brain areas and there were some undirected connections in the corresponding pairs of the AAL brain areas. To eliminate the error of different number of edges in networks, sparsity (S) was introduced to threshold the interregional correlations matrices of the metabolic networks for obtaining binarized matrices, defined as the entire number of edges L divided by the maximum possible number of the edges in a graph [19]. Since there lacks clear standard to choose a single threshold value, so we selected sparsity between 7% and 26% to analyze the small-world properties according to the previous study [22]. In our networks, the maximum possible number of edges was 4005 due to the 90 AAL brain areas. To explore the abnormal interregional correlations and hub regions between APOE *ε*4 carriers and APOE *ε*4 noncarriers, we employed a fixed sparsity (S = 7%) for minimizing the number of false-positive edges [23, 24]. We could obtain the nodes and edges of two networks in an identical number by applying the same sparsity [19].

### Small-world properties analysis

In a recent study, small-world properties of brain metabolic networks supported efficient information transfer at relatively low cost [23]. The mean network clustering coefficient (Cp) and mean network shortest absolute path length (Lp) was used to draw the characterization of the small-world networks. According to the previous study, Ci of the node i is computed by the number of connections between its nearest neighbors divided by these nodes’ possible connections. Cp is defined as the mean of the clustering coefficient over all the nodes in the network [19, 25], which could evaluate the level of the local efficiency of information transfer. The mean shortest absolute path length of node i can be computed by the average of the M-1 shortest absolute path lengths between node i and the other nodes [26]. Lp is defined as the average of the mean shortest absolute path length over all nodes of the network [19].

In previous researches, some criteria were required in networks for finding small-world characteristics:

$$\gamma =C_{p}^{real}\;/\;C_{p}^{random}≻1$$ (1)

$$\lambda =L_{p}^{real}\;/\;L_{p}^{random}\approx 1$$ (2)

Compared with the real network, $C_{p}^{random}$ and $L_{p}^{random}$ indicate Cp and Lp in the matched random networks and these random networks should keep the same number of nodes, edges, and degrees distribution [19, 25].

### Nodal centrality

The node properties of the metabolic networks between APOE *ε*4 carriers and noncarriers were explored with the betweenness centrality of the nodes. In brief, betweenness is defined as the evaluation in the centrality of a node in a network. The betweenness B of node i indicate the entire number of absolute shortest paths between any two other nodes pass through node i[19, 20]. Then, we normalized B_i_ by *b*_*i*_ = *B*_*i*_ / *B*, where B indicates the mean betweenness in networks. Therefore, the hub regions would be generated in many shortest paths between other nodes which have a higher level betweenness and a node i in a network was defined as a hub region if *b*_*i*_ ≥ 2 [19].

### Statistical analysis

To explore the interregional correlations of the metabolic networks in APOE *ε*4 carriers and noncarriers, Fisher’s z transformation was introduced to transform the correlation coefficients into z values and these z values were approximately normally distributed [27]. After comparison of significant between-group differences in interregional correlations, Bonferroni correction was applied with p value of 0.01 to correct for multiple comparisons.

A nonparametric permutation test procedure was applied to test the significant differences in network topology of the metabolic networks such as Cp and Lp. In each fixed sparsity, the two groups’ Cps and Lps were computed, pooled, and divided randomly into 2 randomized groups: APOE *ε*4 carriers and noncarriers. In this process, we kept the number of supposed carriers identical to the number of the original group. To explore the between-group differences of Cp and Lp, we computed the differences in randomized groups and this procedure should be repeated by 1000 times. A same sparsity threshold was applied in every 1000 case and we calculated the properties of each randomized group. Then, the 1000 recorded differences were sorted for finding the between-group differences in real metabolic networks, which were included within 95% (two-tailed) in the supposed between-group differences [19]. If the real between-group difference was outside 95% confidence interval, we considered that the two networks had significant differences. In our study, we selected the sparsity threshold values ranging from 7%<S<26% in this permutation test procedure.

In order to find the differences about the metabolic level in these two groups, we applied the 2-sample t test by using SPM8. Then, we calculated the partial correlation coefficients between FAQ (Functional Assessment Questionnaire) scores and metabolic values in APOE *ε*4 carriers.

## Results

### Comparison in brain metabolism

**Fig 1:**
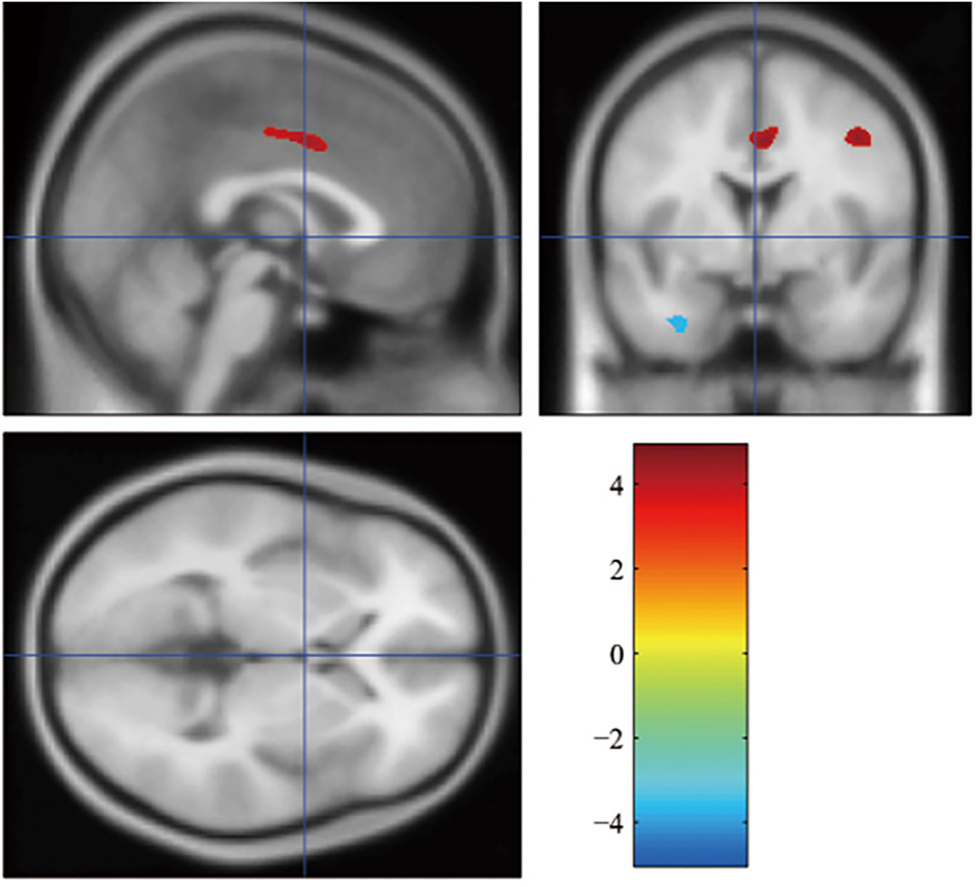
Statistical parametric map showing a higher or lower regional cerebral metabolic rate of glucose (FDR corrected p<0.05) in APOE *ε*4 carriers compared with APOE *ε*4 noncarriers. The color bar indicates the t value. The cluster size > = 5.

In the comparison of the brain metabolism, we used a 2-sample t test (p<0.05, FDR corrected for multiple comparisons) based on a voxel based morphometry. Compared with the noncarriers, the APOE *ε*4 carriers showed decreased metabolic level in parahippocampal gyrus and increased metabolic level in medial frontal gyrus and inferior frontal gyrus (Fig 1).

**Fig 2:**
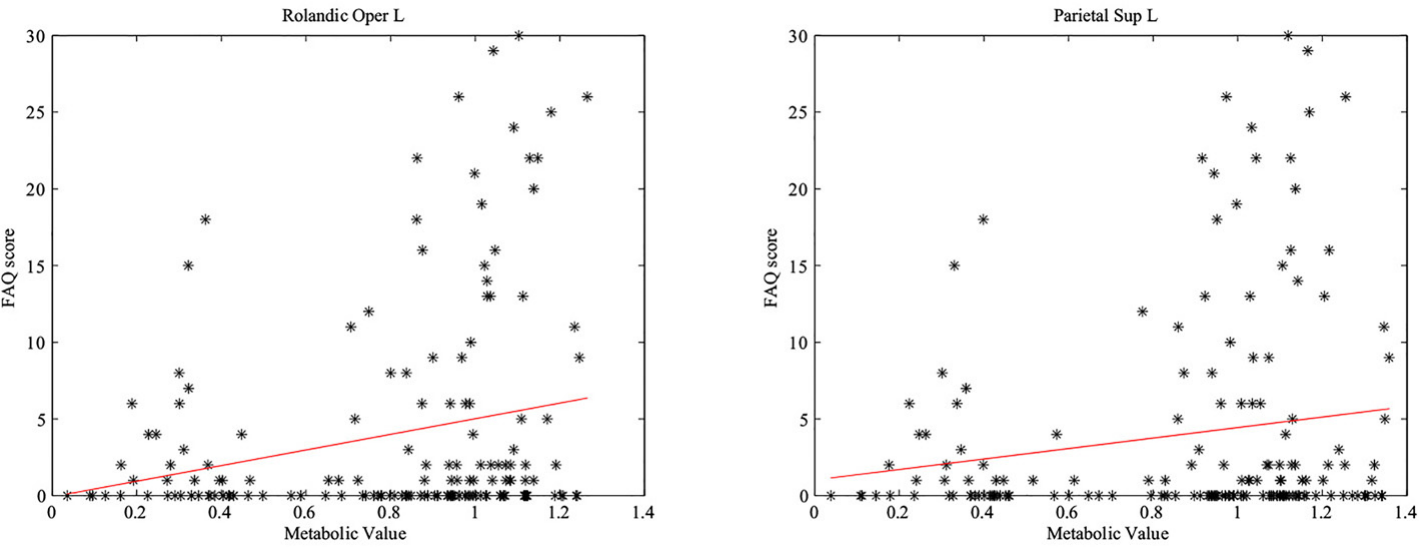
The FAQ scores and the average metabolism in left rolandic operculum and left superior parietal gyrus in APOE *ε*4 carriers.

By computing the partial correlation coefficients between FAQ scores and metabolic values in APOE *ε*4 carriers, we found significant correlations in left rolandic operculum and left superior parietal gyrus. Results are shown in Fig 2.

**Fig 3:**
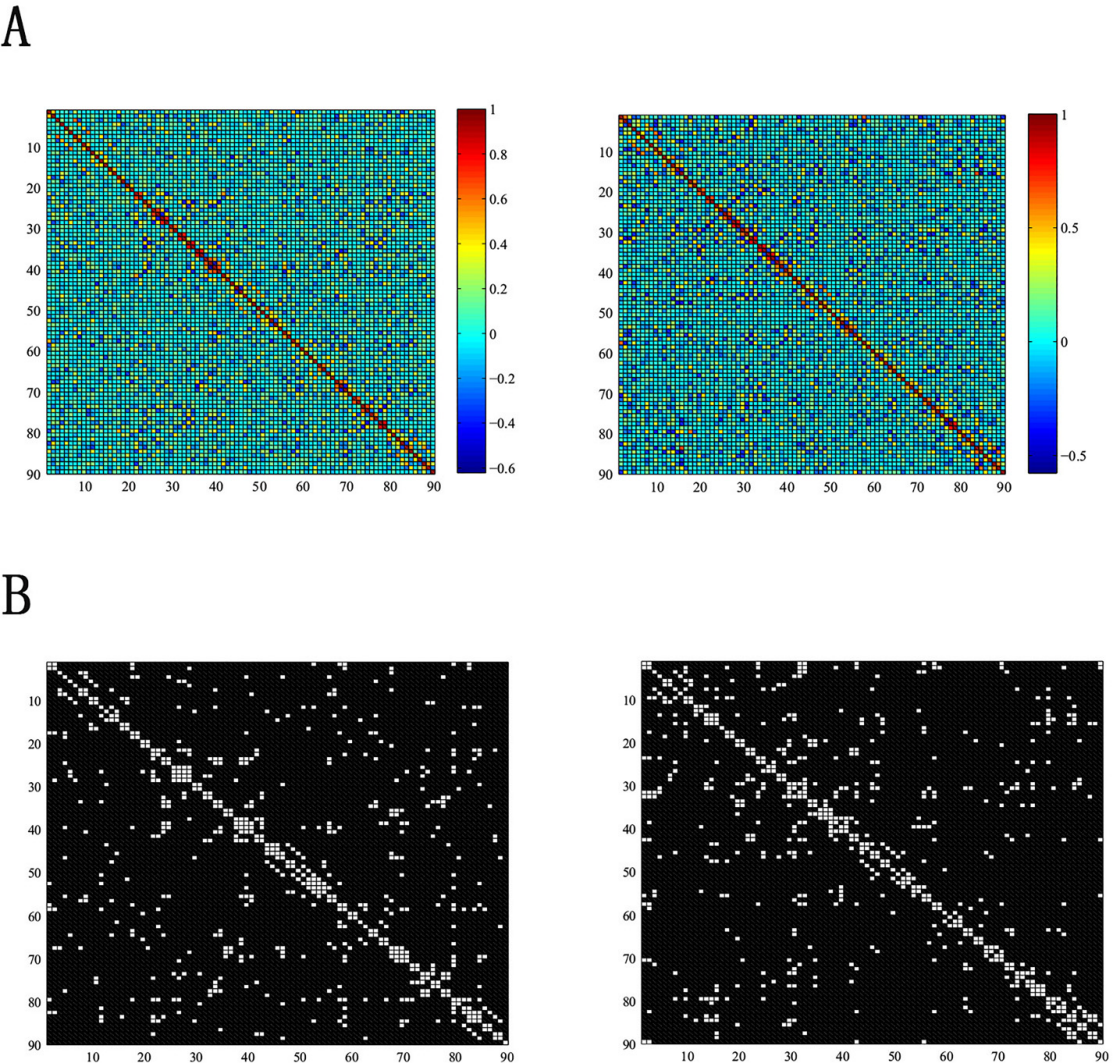
The interregional correlations matrix in APOE *ε*4 carriers and APOE *ε*4 noncarriers. A. The correlations matrices was established by computing the partial correlations between pairs of AAL areas in every group (left: APOE *ε*4 carriers, right: APOE *ε*4 noncarriers) B:The binarized matrices drew by thresholding the partial correlations matrices of A in a sparsity threshold (7%).

The interregional correlation coefficients was computed to establish the correlation matrices (90×90) for APOE *ε*4 carriers and noncarriers. The interregional correlation coefficients were showed in Fig 3.

### Small-world properties of metabolic networks

**Fig 4:**
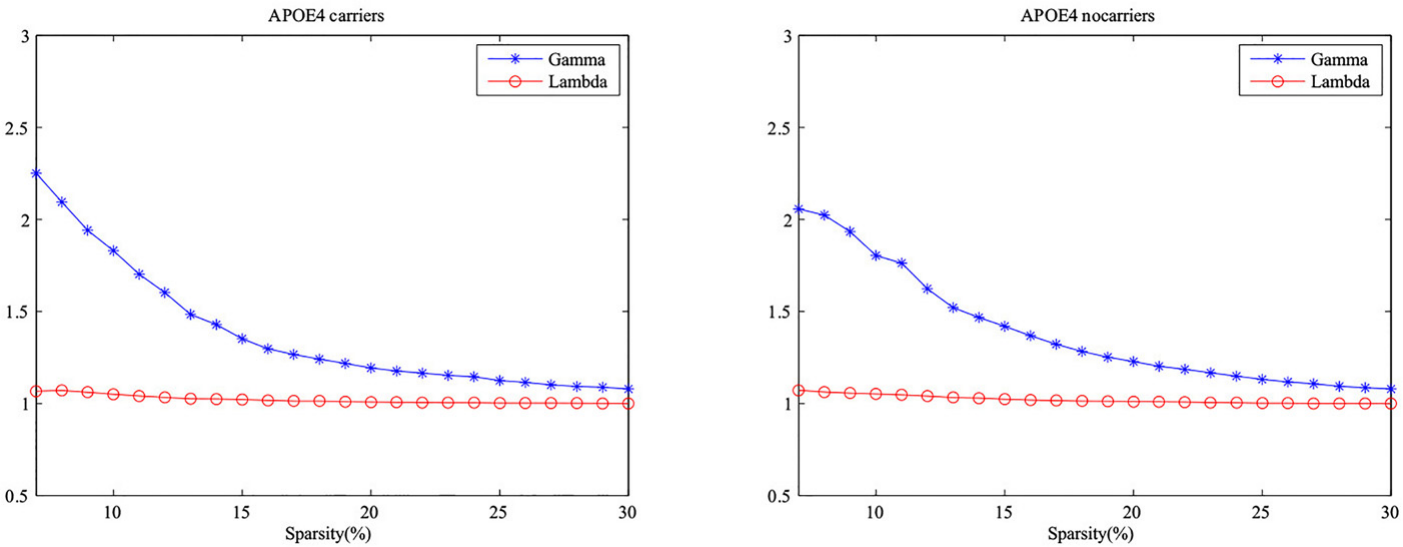
Small-world properties of the metabolic networks. Above graphs indicate the changes in the Gamma $\gamma =C_{p}^{real}\;/\;C_{p}^{random}$ and Lambda $\lambda =L_{p}^{real}\;/\;L_{p}^{random}$ in APOE *ε*4 carriers and APOE *ε*4 noncarriers (sparsity ranging from 7% to 26%).

Relevant studies showed that small-world properties existed in functional networks [15, 23]. We computed the clustering coefficients and absolute path lengths in sparsity threshold values over 7%< S<26% in the two groups. The small-world properties in these groups were shown in Fig 4.

**Fig 5:**
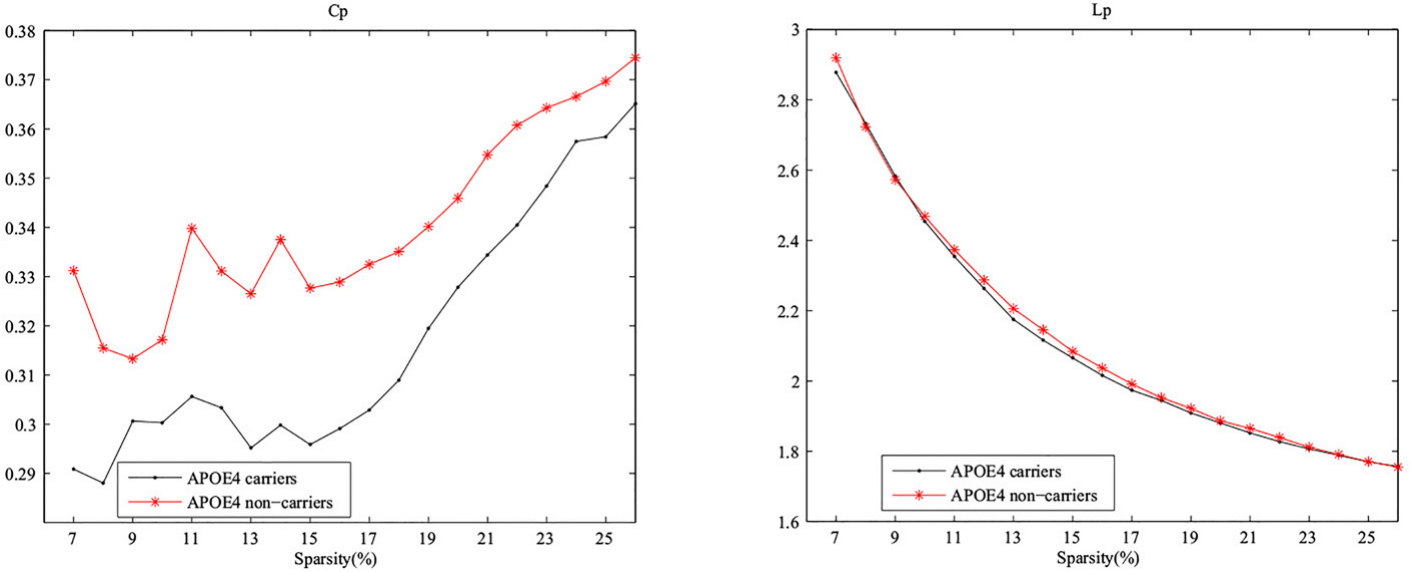
Mean clustering coefficients (Cp) and mean absolute path lengths (Lp) in APOE *ε*4 carriers and APOE *ε*4 noncarriers.

**Fig 6:**
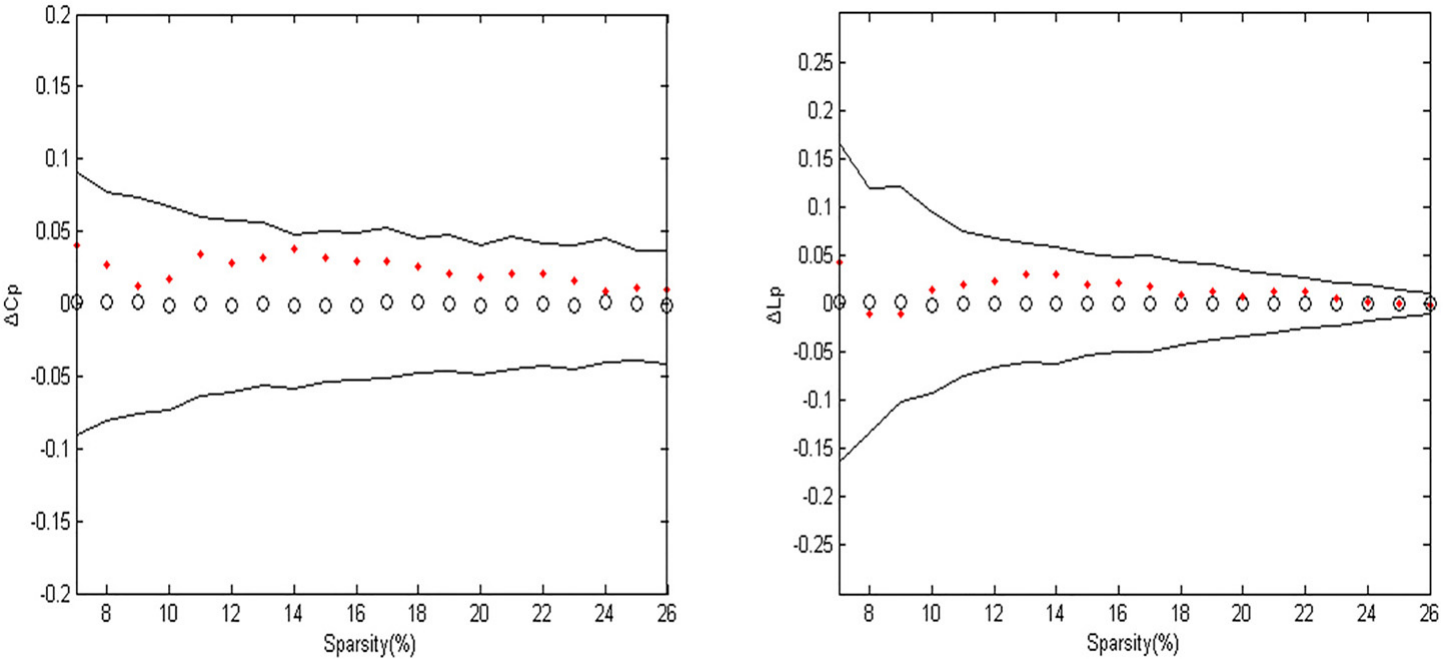
The left image indicates the between-group differences in clustering coefficients (Cp) and the right image indicates the between-group differences in absolute path lengths (Lp). The black open circles show the average values in each property and the black lines indicate the 95% confidence intervals of the between-group differences through 1000 permutation tests in each sparsity.

In the wide range of sparsity, networks both had *γ* > 1 and *λ* ≈ 1, indicating that prominent small-world properties existed in these two groups. From Fig 5, the APOE *ε*4 carriers had a lower level in clustering coefficient and the two groups had the approximately same absolute path length. We applied a permutation test to find the statistically significant between-group differences in these properties (Fig 6). However, no significant difference was found in the clustering coefficients and the absolute path lengths between APOE *ε*4 carriers and noncarriers.

### Hub regions in metabolic networks in APOE *ε*4 carriers

In order to find the between-group differences of the network properties between APOE *ε*4 carriers and noncarriers, a fixed sparsity threshold value was applied (sp = 7%) [13]. The normalized betweenness centrality (*b*_*i*_) in every node was computed to detect the hub regions in the two networks. In the present study, hub nodes were defined if these nodes’ betweenness values were more than twice of the mean betweenness in a network (*b*_*i*_>2). Therefore, some regions were found as hub regions in metabolic networks in each group. The details in hub regions were shown in Table 1.

**Table 1:** Hub regions in metabolic networks of the APOE *ε*4 carriers and the APOE *ε*4 noncarriers listing by the descending order of the APOE *ε*4 carriers’ normalized betweenness.

AAL area	betweenness	
	APOE *ε*4-carriers	APOE *ε*4-noncarriers
Heschl_L	5.68	1.90
Precuneus_L	3.75	1.12
Paracentral_Lobule_L	3.22	0.07
Temporal_Pole_Sup_R	3.05	2.09
Frontal_Med_Orb_R	2.73	0.88
ParaHippocampal_L	2.72	1.22
Rectus_R	2.59	1.19
Cuneus_R	2.31	2.22
Olfactory_L	2.30	0.29
Frontal_Inf_Oper_L	2.01	0.11
Fusiform_L	1.91	2.76
Postcentral_L	1.60	2.65
Cuneus_L	1.33	5.15
Precentral_R	1.20	2.23
Amygdala_R	1.14	2.57
Cingulum_Ant_R	0.85	3.49
Insula_L	0.83	5.11
Pallidum_R	0.54	3.01
Frontal_Inf_Orb_L	0.37	2.12
Frontal_Sup_Medial_R	0.27	2.44
Frontal_Mid_Orb_L	0.20	2.50
Insula_R	0.19	5.77

### Abnormal changes in nodal centrality in APOE *ε*4 carriers

**Fig 7:**
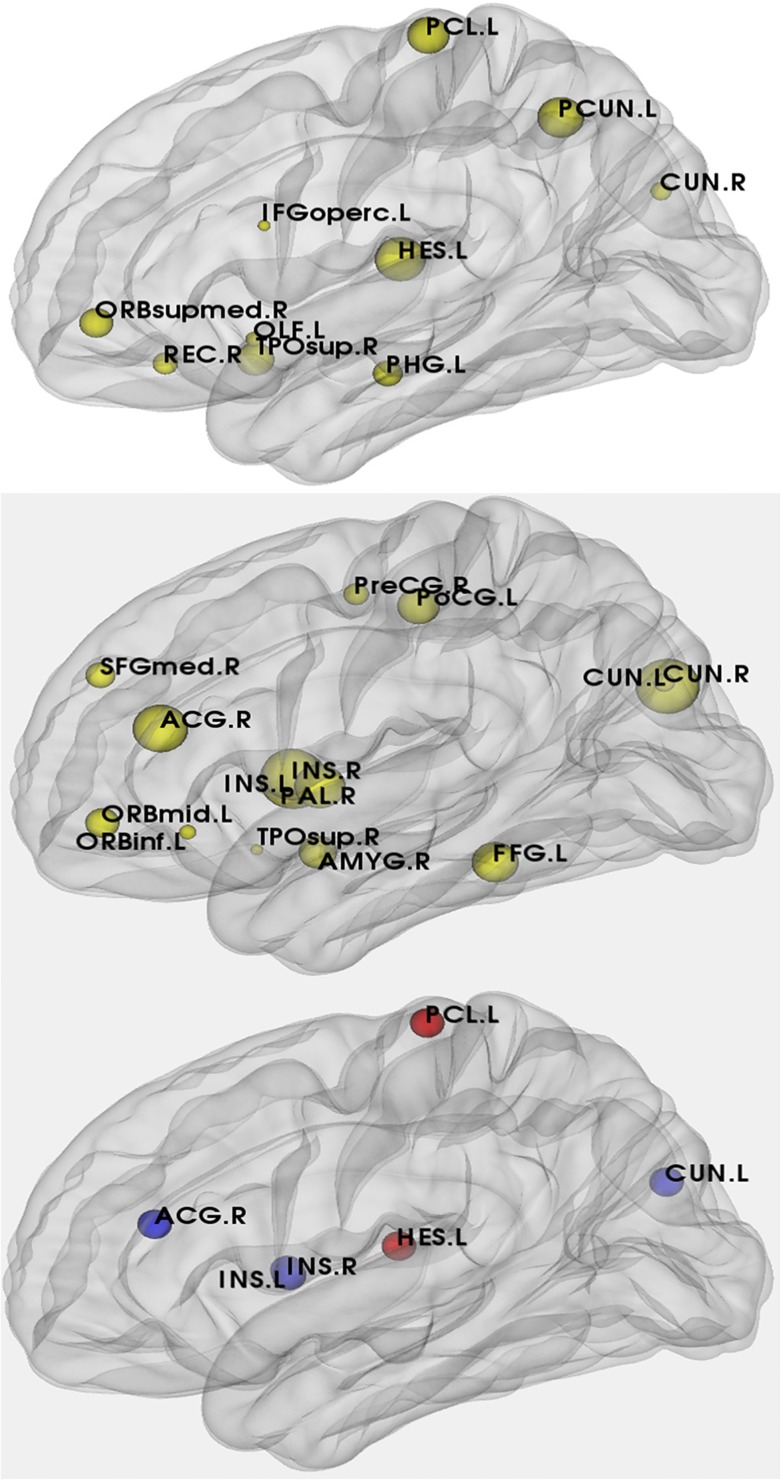
A. The hub regions in APOE *ε*4 carriers (The size of the circles represents the nodal centrality in this brain region). B. The hub regions in APOE *ε*4 noncarriers. C. Differences of between-group nodal centrality in the APOE *ε*4 carriers compared with the APOE *ε*4 noncarriers. The six hub regions which are at least in a network of the APOE *ε*4 carriers and the APOE *ε*4 noncarriers and these hub regions indicate the significant between-group differences(p<0.05). The blue spheres represent nodal centrality with significant decreases and the red spheres represent nodal centrality with significant increases in the APOE *ε*4 carriers compared with the APOE *ε*4 noncarriers.

In our study, we applied 1000 nonparametric permutation tests to detect the between-group differences in nodal centrality. The abnormal changes in nodal centrality in the two groups were shown in Fig 7. Right superior temporal gyrus and right cuneus were the common hub regions in the two groups. Compared with the APOE *ε*4 noncarriers, the nodal centralities of the APOE *ε*4 carriers showed significant decreases in left insula, right insula, right anterior cingulate, right paracingulate gyri, left cuneus and significant increases in left paracentral lobule and left heschl gyrus.

### Abnormal changes in interregional correlations in APOE *ε*4 carriers

**Fig 8:**
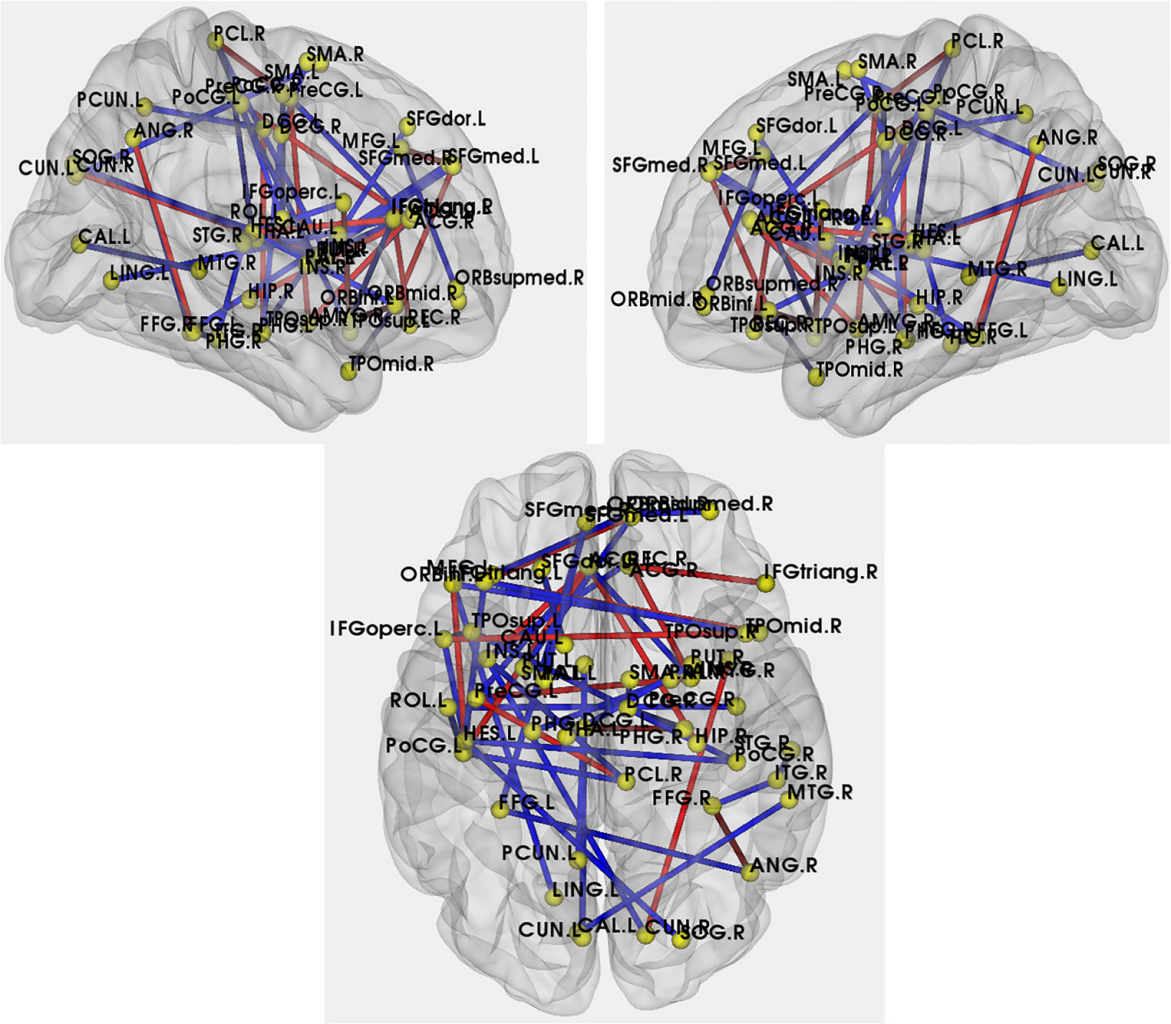
Abnormal interregional correlations in APOE *ε*4 carriers compared with APOE *ε*4 noncarriers. The yellow dots indicate the AAL brain regions which showed the significantly abnormal correlations. The red and blue lines indicate the significantly increased and decreased interregional correlations.

To find the between-group differences in correlation coefficients, the Fisher’s z transformation was utilized. The abnormal interregional correlations were shown in Fig 8.

In Fig 8, a decreased long distance interregional correlation existed between right lenticular nucleus and left parahippocampal gyrus. Additionally, some increased short distance interregional correlations existed in prefrontal cortex.

## Discussion

In prior studies, the FDG-PET metabolic networks were established by computing correlation coefficients between pairs of the brain regions. Essential to this effort is the cerebral metabolic level that could contribute to brain network researches [15, 20, 28]. Gretel *et al*. analyzed the abnormal changes by using the brain glucose metabolism co-variations in Alzheimer disease (AD) and Mild Cognitive Impairment (MCI) [20]. Besides, a study in 2014 demonstrated that APOE *ε*4 genotype was associated with the regional glucose hypometabolism [29]. In this study, we explored the metabolic network in APOE *ε*4 carriers compared with the noncarriers. Our findings could be summarized as follows: 1. The metabolic networks of the APOE *ε*4 carriers and noncarriers both showed small-world characteristics. 2. Abnormal nodal centrality changes were found in APOE *ε*4 carriers. 3. Significant abnormities of interregional correlations were detected in APOE *ε*4 carriers. These results indicate that APOE *ε*4 carriers have some similar aspects as AD patients and APOE *ε*4 could be a risk factor for AD.

### Comparison in brain metabolism

To find differences in brain metabolism, 2-sample t test (p<0.05, FDR corrected for multiple comparisons) was utilized on a voxel based morphometry. As is shown in Fig 1, the APOE *ε*4 carriers showed decreased metabolic level in parahippocampal gyrus and increased metabolic level in medial frontal gyrus and inferior frontal gyrus compared with the noncarriers. The decrease of metabolism in parahippocampal gyrus was coherent with the previous study, reporting the lower regional glucose metabolism in parahippocampal gyrus in AD with APOE *ε*4 [30]. In addition, subjects with subjective memory complaints also showed reduced glucose metabolism in parahippocampal gyrus [31]. This decrease indicates that APOE *ε*4 may contribute to the declines of brain glucose metabolism in parahippocampal gyrus, which might lead to the memory impairment [30]. And the activation in frontal lobe was improved might be a response to medication [32].

### Correlation analysis in FAQ scores

We computed partial correlation coefficients between the FAQ scores and the metabolism in APOE *ε*4 carriers. FAQ is a bounded outcome with 0 scored as “no impairment” and 30 scored as “severely impaired” [33]. Fig 2 showed the negative correlations between FAQ scores and the mean metabolic values in left rolandic operculum and left superior parietal gyrus in the APOE *ε*4 carriers. Left rolandic operculum was thought to play a role in both sentence-level and local syntactic encoding of speaking [34] and left superior parietal gyrus might be related to the activation in the conjunction task of spatial attention [35]. Accordingly, the negative correlations might indicate the declines of cognitive and memory in APOE *ε*4 carriers [36, 37].

### Small-world properties in networks

In the previous researches, small-world properties were detected in the complex brain metabolic networks [15, 23, 38]. Fig 4 showed that both networks in APOE *ε*4 carriers and noncarriers had the small-world properties. It also can be observed in Figs 5 and 6 that these two groups presented no statistically significant differences in absolute path length and clustering coefficient after 1000 permutation test, indicating the two groups had no significant between-group difference in local efficiency of information transfer.

### Abnormal changes in nodal centrality in APOE *ε*4 carriers

From Fig 7, we can see that the nodal centralities of the APOE *ε*4 carriers showed significant decreases in left insula, right insula, right anterior cingulate, right paracingulate gyri, left cuneus and significant increases in left paracentral lobule and left heschl gyrus compared with noncarriers. Kim *et al*. described rCBF (Regional Cerebral Perfusion) of the APOE *ε*4 carriers (both in normal controls and AD patients) had a significant decrease in insula [39]. And Ewers *et al*. and Forster *et al*. detected reduced cerebral glucose metabolism in anterior superior insula and right insula in AD [40, 41]. Since insula has been interpreted as a well-established role in visceral sensory area and emotional processing area [42–44], the reduced nodal centrality in insula may be related to the poor performance of APOE *ε*4 carriers in cognition [45]. The prior study indicated that the APOE *ε*4 carriers had significant decreases in cerebral metabolic rate for glucose (CMRgl) in cingulate cortex and parahippocampal, which was associated with the predisposition to Alzheimer’s dementia [46]. Thus, the abnormal decreases in nodal centrality in cingulate and paracingulate gyri may be a support to the point that APOE *ε*4 was a risk factor for AD. We also detected a decreasing nodal centrality in left cuneus of APOE *ε*4 carriers compared with noncarriers and cuneus was a role in visual information transfer [47]. Similarly, a study on the PET patterns of brain activation reported that there were notable declines in activation of APOE *ε*4 carriers in left cuneus. So APOE genotype may work for cerebral physiologic activity [48]. In addition to these brain regions with lower nodal centralities, two brain regions with higher nodal centralities existed in APOE *ε*4 carriers, in comparison with noncarriers. Heschl gyrus was considered as an essential area in musical aptitude [49]. Recent research also reported heschl gyrus, as a marker for human primary auditory cortex, was included in auditory network (AUN)[50]. The increased nodal centrality of heschl gyrus may be related to the abnormal changes in connectivity between AUN and other regions in frontal and parietal lobes [51]. So the APOE *ε*4 may have a negative influence on the connectivity of AUN and other networks [52]. The previous studies reported that APOE *ε*4 carriers had the similar pattern as AD [46] and the paracentral lobule was the hub regions in AD [19]. The paracentral lobule was located in association cortex which was thought to mediate a variety of cognitive functions [53]. The increased nodal centrality of paracentral lobule in our results may relate to the increased within-lobe functional connectivity in AD patients, which may be characterized as compensatory recruitment in APOE *ε*4 carriers [54–57].

### Altered interregional correlations in APOE *ε*4 carriers

The details of the abnormal interregional correlations in the APOE4 *ε*4 carriers were shown in Fig 8. Compared with the APOE *ε*4 noncarriers, 39 decreased interregional correlations and 9 increased interregional correlations were shown in APOE *ε*4 carriers. In prior studies of APOE-related changes, the default-mode synchronization was associated with the genetic vulnerability [58]. Furthermore, decreased resting-state activity of hippocampus may suggest the disrupted connectivity of PET researches in course of early AD [59]. In our study, a decreased long distance interregional correlation existed between right lenticular nucleus and left parahippocampal gyrus. Moreover, there was a decreased interregional correlation between left median cingulate and right parahippocampal gyrus. These disrupted interregional correlations may lead to the increase of the cost in information transfer in the human brain and account for less activation in hippocampus during encoding in the APOE *ε*4 carriers [60]. Aligned with the resting-state fMRI study, our results also found decreased correlations between the prefrontal and parietal lobes in APOE *ε*4 carriers, in contrast with noncarriers [56]. These decreased interregional correlations may lead to a lower global efficiency and the restriction efficiency of information processing in APOE *ε*4 carriers [61]. Additionally, we found some increased short distance interregional correlations in prefrontal cortex. Consistent with the increased activities in prefrontal detected by Kun Wang *et al*., these increased short distance interregional correlations might be considered as a compensatory recruitment of cognitive resources among AD [56], which indicated APOE *ε*4 carriers with genetic risk for AD, needing additional support to achieve a normal cognitive level [62, 63].

## Conclusion

In conclusion, this paper tried to investigate the between-group differences of network properties by constructing the metabolic network in the APOE4 *ε*4 carriers and noncarriers. Compared with the noncarriers, the APOE *ε*4 carriers showed decreased metabolic level in parahippocampal gyrus, as well as increased metabolic level in medial frontal gyrus and inferior frontal gyrus. The metabolic networks of the APOE *ε*4 carriers and noncarriers both indicated the small-world properties. Between-group nodal centralities with significantly abnormal changes were observed. In addition, the disrupted long distance interregional correlations were discovered in APOE *ε*4 carriers, which might cause the increase of the cost in information transfer. Furthermore, the organization of metabolic network in APOE *ε*4 carriers indicated a less optimal pattern,suggesting that APOE *ε*4 genotype might be a risk factor for AD.

## Methodological Limitations

The limitations of our study lie in two aspects. Firstly, for statistical significance in large sample, all subjects including MCIs, ADs and health controls were gathered together to form the APOE *ε*4 carriers and noncarriers group. Unfortunately, according to the previous studies, altered topological organization may exist in the AD or MCI patients, which may affect the accuracy of our results to some extents. Secondly, our study only focuses on the global network manifestation of brain malfunction in APOE genotype carriers indicated by the metabolic connectivity. Further studies should be addressed on the multi-level network features in brain imaging.

## Supporting Information

S1 File ADNI Acknowledgment List. (PDF)
